# MRI monitoring of USPIO-labeled BMSCs combined with alginate scaffold for cartilage defect repair

**DOI:** 10.3389/fbioe.2025.1554292

**Published:** 2025-03-17

**Authors:** Shanyu Lu, Zhenyu Liu, Meiling Qi, Haocheng Zhen, Jing Luo, Yingchao Wang, Le Chang, Xiaolong Bai, Yingguang Jiao, Xinyao Chen, Junping Zhen

**Affiliations:** ^1^ College of Medical Imaging, Shanxi Medical University, Taiyuan, Shanxi, China; ^2^ Department of Imaging, Second Hospital of Shanxi Medical University, Taiyuan, Shanxi, China; ^3^ Clinical and Basic Medical College, Shandong First Medical University, Jinan, Shandong, China; ^4^ Shanxi Key Laboratory for Immunomicroecology, Taiyuan, Shanxi, China; ^5^ Molecular Imaging Laboratory, Second Hospital of Shanxi Medical University, Taiyuan, Shanxi, China

**Keywords:** cartilage repair, T2 mapping MRI, USPIO, bone marrow mesenchymal stem cells, non-invasive monitoring

## Abstract

**Objective:**

This study aimed to evaluate the effectiveness of bone marrow mesenchymal stem cells (BMSCs) combined with sodium alginate scaffolds in repairing knee cartilage defects in New Zealand rabbits. Additionally, it assessed the potential of functional magnetic resonance imaging (fMRI) for non-invasive monitoring of the dynamic repair process.

**Methods:**

Rabbits were randomly divided into four groups: Group A (control), Group B (sodium alginate scaffold), Group C (BMSCs-sodium alginate scaffold), and Group D (USPIO-labeled BMSCs-sodium alginate scaffold). A cartilage defect model was created, and the respective materials were implanted into the defect regions. T2 mapping MRI was performed at weeks 1, 2, and 4 post-surgery to evaluate the repair process, followed by histological analysis to confirm the outcomes.

**Results:**

BMSCs significantly promoted cartilage defect repair and accelerated the degradation of sodium alginate scaffolds. Macroscopic and histological evaluations revealed repair tissue formation in Groups C and D by week 1, with most defect regions filled with new cartilage by week 4. T2 mapping analysis showed a gradual decline in T2 values in Group B, a more pronounced decrease in Group C, and consistently lower T2 values in Group D compared to Group C, with a slow upward trend over time.

**Conclusion:**

This study demonstrated that BMSCs exhibit significant regenerative potential for cartilage defect repair. USPIO labeling enables non-invasive, dynamic monitoring of the repair process without adverse effects on cell viability or differentiation. These findings provide experimental evidence supporting the application of BMSCs combined with magnetic labeling technology in cartilage regeneration.

## 1 Introduction

Osteochondral defects caused by degenerative arthritis, inflammatory arthritis, or trauma are among the leading causes of chronic joint pain and disability (Hunter, 1995; Messner and Maletius, 1996; Lespasio et al., 2017; Katz et al., 2021). The regenerative capacity of cartilage tissue is inherently limited due to its avascular nature and low cellular density (Kim et al., 2020; Guo et al., 2023). While current therapeutic strategies, including pharmacological treatments, physical therapy, and surgical interventions, can somewhat alleviate symptoms, they are significantly limited in achieving complete cartilage repair (Abdel-Aziz et al., 2021). Consequently, there is an urgent need to develop novel and effective strategies for joint repair.

Bone marrow mesenchymal stem cells (BMSCs), with their remarkable self-renewal capability and multipotent differentiation potential, have emerged as a key cell type for cartilage regeneration research (Delplace et al., 2021). Studies have demonstrated that BMSCs can effectively promote chondrocyte proliferation and differentiation, thereby accelerating the repair of cartilage defects (Oh et al., 2018; Selvasandran et al., 2018; Kim et al., 2019). However, the successful transplantation of BMSCs requires a scaffold material that provides a suitable microenvironment to support cell growth, proliferation, and differentiation (Huey et al., 2012). Sodium alginate, a natural biomaterial with excellent biocompatibility and biodegradability, has been widely applied in tissue engineering (García-Couce et al., 2021; Hu et al., 2023). It is an ideal scaffold for cell adhesion and mimics the extracellular matrix, creating favorable conditions for BMSC proliferation and differentiation (Dhamecha et al., 2019; Cattelan et al., 2020; Yuan et al., 2020). Therefore, selecting an appropriate scaffold is critical for achieving effective cell implantation and tissue repair.

Moreover, non-invasive monitoring of the repair process is essential in cartilage regeneration research (Bulte and Daldrup-Link, 2018). Magnetic resonance imaging (MRI) has become the preferred technique for monitoring cartilage repair due to its lack of ionizing radiation, high resolution, and superior soft tissue contrast (Ronga et al., 2014; Nieminen et al., 2022; Zibetti et al., 2023). Among MRI techniques, T2 mapping is particularly effective for quantitatively assessing changes in water content and collagen fiber organization within cartilage, making it a valuable tool for detecting early cartilage degeneration and evaluating repair outcomes (Andreisek and Weiger, 2014; Chen et al., 2018; Banitalebi et al., 2021). Additionally, ultrasmall superparamagnetic iron oxide (USPIO) nanoparticles, as MRI-based cell tracking agents, enable dynamic monitoring of BMSCs during cartilage repair.

This study aims to systematically investigate the repair potential of BMSCs in a cartilage defect model, evaluate the role of sodium alginate in cartilage regeneration, and assess its feasibility and effectiveness as a scaffold material. Furthermore, T2 mapping was utilized to quantitatively monitor the cartilage repair process, while USPIO-labeled BMSCs were used for *in vivo* cell tracking to non-invasively monitor cell survival and transplantation outcomes. Through this research, we aim to provide experimental evidence for applying BMSCs and sodium alginate in cartilage repair and explore the potential of MRI as a non-invasive monitoring tool for cartilage regeneration, laying a scientific foundation for future clinical practice.

## 2 Materials and methods

### 2.1 Experimental animals

Four healthy 3–4-week-old New Zealand white rabbits (weighing approximately 220 g) and 24 healthy 4-month-old New Zealand white rabbits (weighing approximately 2 kg) were obtained from the Animal Center of Shanxi Medical University. All experimental procedures adhered to ethical guidelines for animal research (License No. SCXK (Jin) 2019-0005, Ethical Approval No. DW2023063).

### 2.2 BMSC culture and labeling

#### 2.2.1 Isolation and culture of BMSCs

Four 3–4-week-old rabbits were randomly divided into experimental and control groups (n = 2 per group). The experimental group received an auricular vein injection of 25 mg Fe/kg USPIO, while the control group received no treatment. Two days later, both groups were euthanized, immersed in 75% ethanol for 10 min, and their femurs and tibias were harvested and stored in PBS.

In a sterile environment, the bone marrow cavity was repeatedly flushed with DMEM/F12 medium using a 1 mL syringe. The flushed suspension was filtered through a 100 µm mesh to remove large tissue debris and clots. The filtrate was seeded into complete medium containing 10% fetal bovine serum and 1% penicillin-streptomycin and incubated at 37°C in 5% CO_2_. The medium was replaced after 48 h and subsequently every 3 days.

#### 2.2.2 Identification of BMSCs by immunofluorescence

BMSCs were digested, resuspended, and seeded at a density of 200,000 cells per well on six-well plate coverslips. After overnight incubation at 37°C and 5% CO_2_, cells were washed with PBS, fixed with 4% paraformaldehyde, and blocked with goat serum. Primary antibodies (CD90, CD105, and CD45) were added and incubated overnight at 4°C in a humid chamber. The next day, cells were washed with PBS, incubated with Cy3-conjugated secondary antibodies for 1 h in the dark, stained with DAPI, and mounted. Cell phenotypes were observed and recorded using a fluorescence microscope.

#### 2.2.3 Proliferation assay of BMSCs

When BMSCs reached 80% confluence, they were digested with 0.25% trypsin and resuspended to prepare a single-cell suspension. The suspension was seeded into 96-well plates at a concentration of 2 × 10^3^ cells per well in 100 µL medium. After adding 10 µL enhanced CCK-8 reagent to each well, plates were incubated at 37°C for 2 h. Absorbance values (OD) at 450 nm were measured using a microplate reader, and measurements were repeated daily for 7 days to construct proliferation curves.

#### 2.2.4 Chondrogenic differentiation of BMSCs

BMSCs at 80%–90% confluence were digested with 0.25% trypsin, resuspended in a semi-complete medium at 7.5 × 10^5^ cells/mL, and centrifuged for 5 min to remove the supernatant. Cells were resuspended in a chondrogenic induction medium at 5 × 10^5^ cells/mL, and 1 mL of suspension was transferred into 15 mL centrifuge tubes and centrifuged. The pellets were incubated in a chondrogenic induction medium, which was replaced every 2–3 days. After 3–4 weeks, samples were fixed, embedded, sectioned, and stained with Alcian blue for microscopic observation of cartilage nodule formation.

### 2.3 Scaffold preparation

#### 2.3.1 Preparation of sodium alginate gel

A 1.2% (w/v) sodium alginate solution was prepared and aliquoted into three 15 mL centrifuge tubes. The first tube was untreated, the second was supplemented with BMSCs at 5.8 × 10^6^ cells/mL, and the third contained USPIO-labeled BMSCs at the same density.

From each tube, 25 µL gel solution was added dropwise into 1.13% CaCl_2_ solution and incubated for 10 min to form gel microspheres. These included pure sodium alginate gel, BMSCs–sodium alginate gel, and USPIO-labeled BMSCs–sodium alginate gel. The microspheres were stored in a complete medium for later use.

### 2.4 Experimental design and grouping

#### 2.4.1 Grouping

Twenty-four 4-month-old rabbits were randomly assigned to four groups (n = 6 per group). A cylindrical cartilage defect (3 mm diameter, 2 mm depth) was created in the femoral trochlear surface of the right knee. Groups were treated as follows: Group A: Control, no material implanted. Group B: Sodium alginate, pure sodium alginate gel implanted. Group C: Stem cell, BMSCs–sodium alginate gel implanted. Group D: USPIO-labeled stem cell, USPIO-labeled BMSCs–sodium alginate gel implanted.

#### 2.4.2 Surgical procedure

Rabbits were anesthetized with an intramuscular injection of 0.1–0.2 mL/kg Su-Mianxin II. The right knee was shaved and disinfected with iodophor. The patella was medially dislocated, and the fascia, ligament, and fat pad were exposed to access the femoral trochlear groove. After creating the cartilage defect, the corresponding material was implanted, the joint capsule was closed, and tissues were sutured.

### 2.5 MRI monitoring

#### 2.5.1 T2 mapping parameters

T2 mapping of the right knee joint was performed at 1, 2, and 4 weeks post-surgery using a GE 3.0T MRI scanner. Scan parameters included: TR = 1700 ms; TE = 14.4, 28.8, 43.3, 57.7, 72.1, 86.5, 101.0, and 115.4 ms (8 echoes); slice thickness = 1.5 mm; interslice gap = 0.2 mm; FOV = 80 × 80 mm; matrix = 256 × 256; NEX = 2.

**FIGURE 1:**
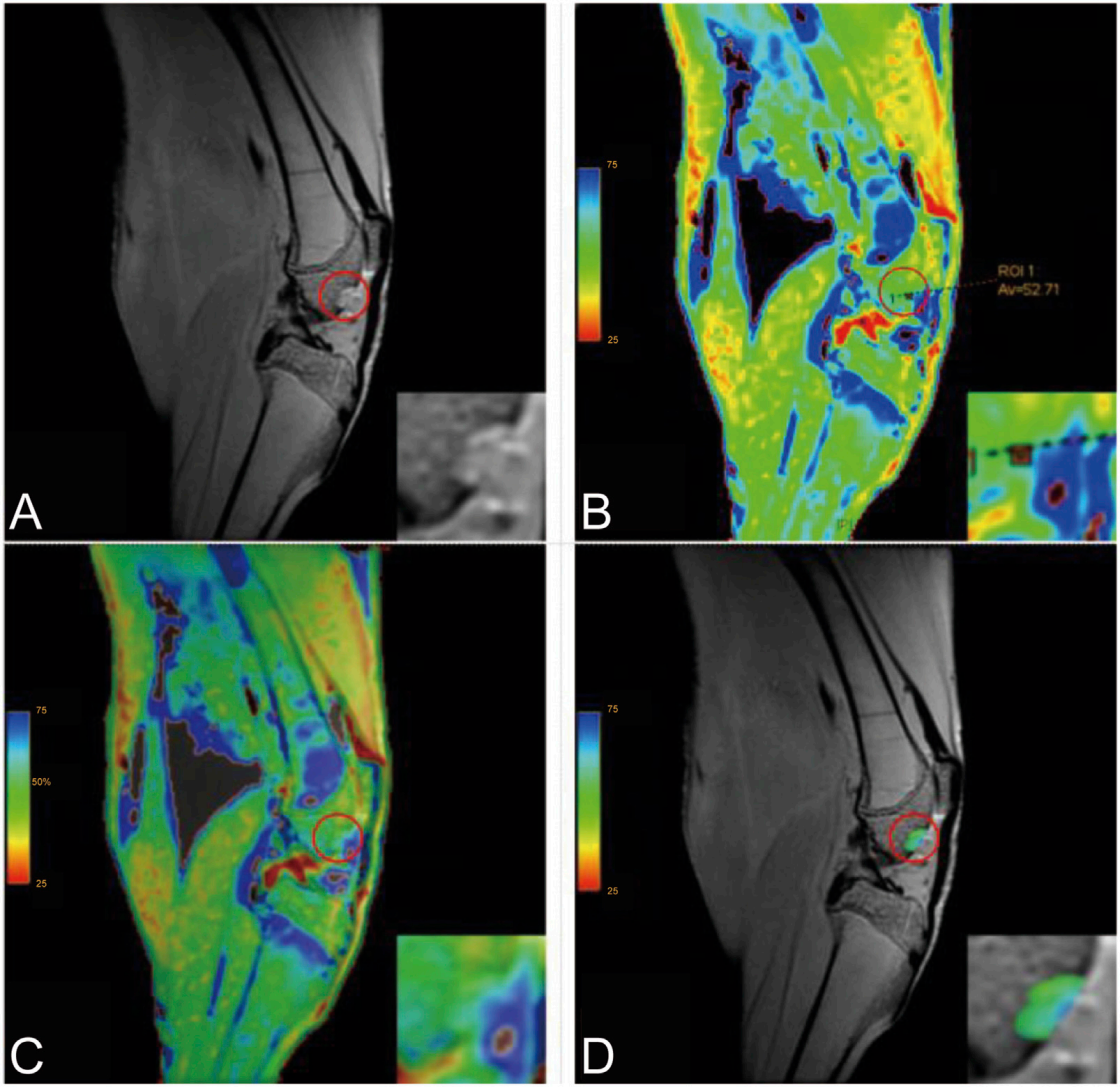
Post-Processing of T2 Mapping (Inset: Magnified View). **(A)** Original image; **(B)** Post-processed image showing an ROI T2 value of 52.71; **(C)** Overlay image; **(D)** ROI overlay image.

Imaging data were analyzed using Functool software on an AW554.6 workstation by two experienced MRI radiologists. Regions of interest (ROIs) were manually delineated, with three measurements per knee averaged. Inter-rater reliability was evaluated using intraclass correlation coefficients (ICCs). The processing workflow is provided as Figure 1.

### 2.6 Data collection and analysis

#### 2.6.1 Macroscopic and histological assessment

At 1, 2, and 4 weeks post-surgery, two rabbits from each group were euthanized for gross specimen observation and cartilage repair assessment. Specimens were fixed, decalcified, embedded, sectioned, and stained with HE, Safranin O–fast green, and toluidine blue for histological evaluation.

#### 2.6.2 Statistical analysis

Data were analyzed using SPSS 27.0. Normally distributed quantitative data were expressed as mean ± standard deviation. Group comparisons were conducted using independent sample t-tests, with Welch’s test applied for unequal variances. Statistical significance was defined as P < 0.05.

## 3 Results

### 3.1 Morphology and identification of BMSCs

#### 3.1.1 Cell morphology

**FIGURE 2:**
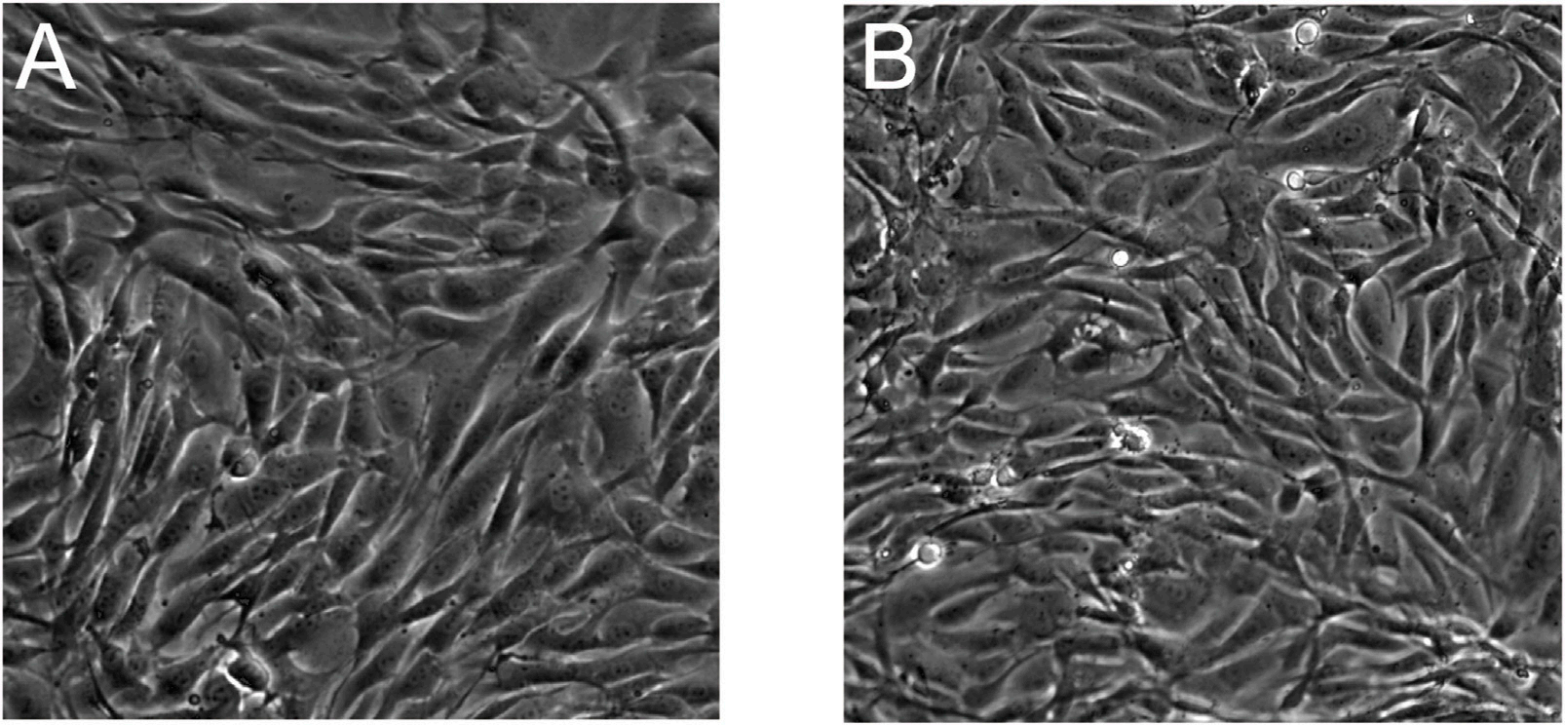
Morphology of BMSCs observed under a microscope. **(A)** Unlabeled BMSCs at ×20 magnification; **(B)** USPIO-labeled BMSCs at ×20 magnification. Both groups exhibit spindle-shaped adherent growth patterns with no discernible morphological differences between the labeled and unlabeled cells, indicating that USPIO labeling does not alter cell morphology.

During the cell culture process, both USPIO-labeled and unlabeled BMSCs exhibited elongated spindle-shaped adherent growth, with cells densely arranged and minimal suspended cells. Microscopic observation revealed comparable growth rates between the two groups (Figure 2).

#### 3.1.2 Identification of BMSCs via immunofluorescence

**FIGURE 3:**
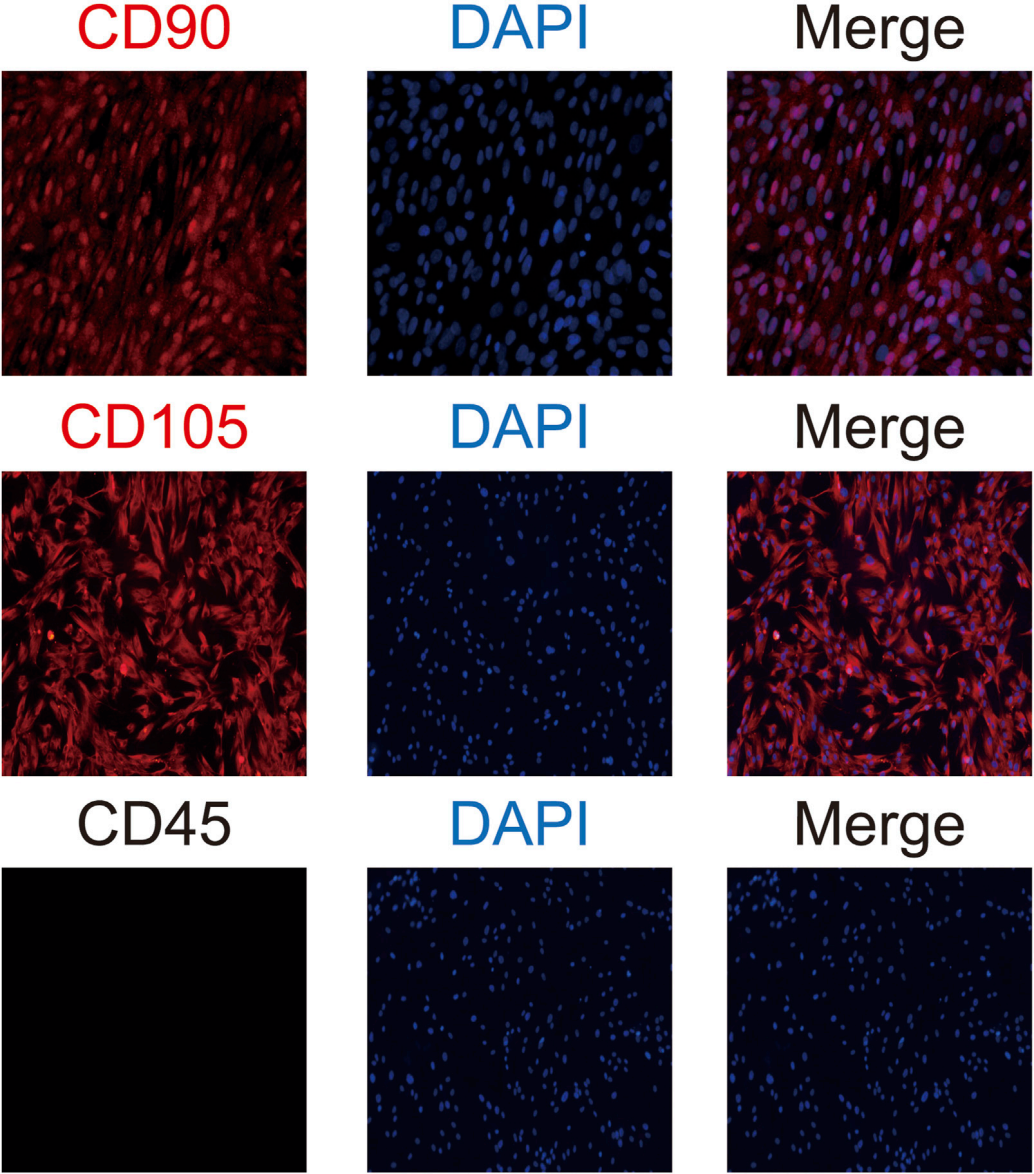
Immunofluorescence Identification of BMSCs. Immunofluorescence analysis of BMSC surface markers demonstrates positive expression of CD90 and CD105 (red) with a positivity rate exceeding 90%, while nuclei are counterstained with DAPI (blue). CD45 expression is negative, confirming the absence of hematopoietic origin. The merged images illustrate the co-localization of CD90 and CD105 with nuclei, with no detectable fluorescence for CD45, further validating the cells as BMSCs.

Immunofluorescence analysis of surface markers revealed that BMSCs expressed CD90 and CD105 with positivity rates exceeding 90%, while CD45 was negative (Figure 3). These findings confirm the cells’ identity as BMSCs.

#### 3.1.3 Assessment of proliferation capacity

**FIGURE 4:**
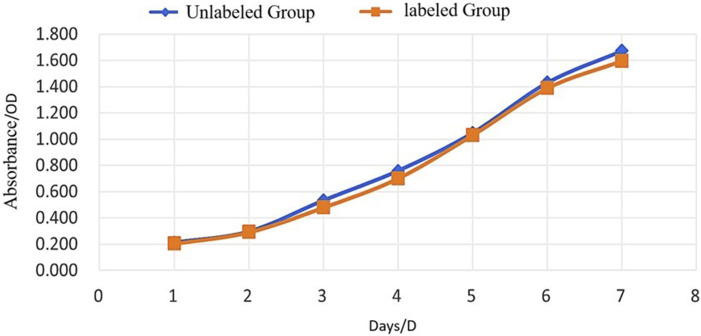
Proliferation of Unlabeled and USPIO-Labeled BMSCs Over Time. Growth curves of BMSCs assessed by optical density (OD) at 450 nm over 7 days. Both the unlabeled group (blue) and the USPIO-labeled group (orange) exhibit comparable proliferation rates with no statistically significant differences (P > 0.05). These results indicate that USPIO labeling does not impair the proliferative capacity of BMSCs.

Cell proliferation was evaluated by measuring optical density (OD) at 450 nm using a microplate reader over seven consecutive days. The proliferation curves of USPIO-labeled and unlabeled BMSCs were nearly identical, with no statistically significant differences (P > 0.05, Figure 4; Table 1). These results indicate that USPIO labeling at a concentration of 25 mg Fe/kg does not significantly affect the proliferation capacity of BMSCs.

**TABLE 1:** Optical density (OD) values of USPIO-labeled and unlabeled BMSCs over seven days.

Days	1d	2d	3d	4d	5d	6d	7d
Unlabeled group	0.21 ± 0.01	0.30 ± 0.01	0.53 ± 0.03	0.76 ± 0.02	1.05 ± 0.02	1.43 ± 0.10	1.67 ± 0.08
Labeled group	0.20 ± 0.01	0.29 ± 0.02	0.48 ± 0.02	0.70 ± 0.09	1.03 ± 0.02	1.39 ± 0.08	1.60 ± 0.15

#### 3.1.4 Evaluation of chondrogenic differentiation capacity

**FIGURE 5:**
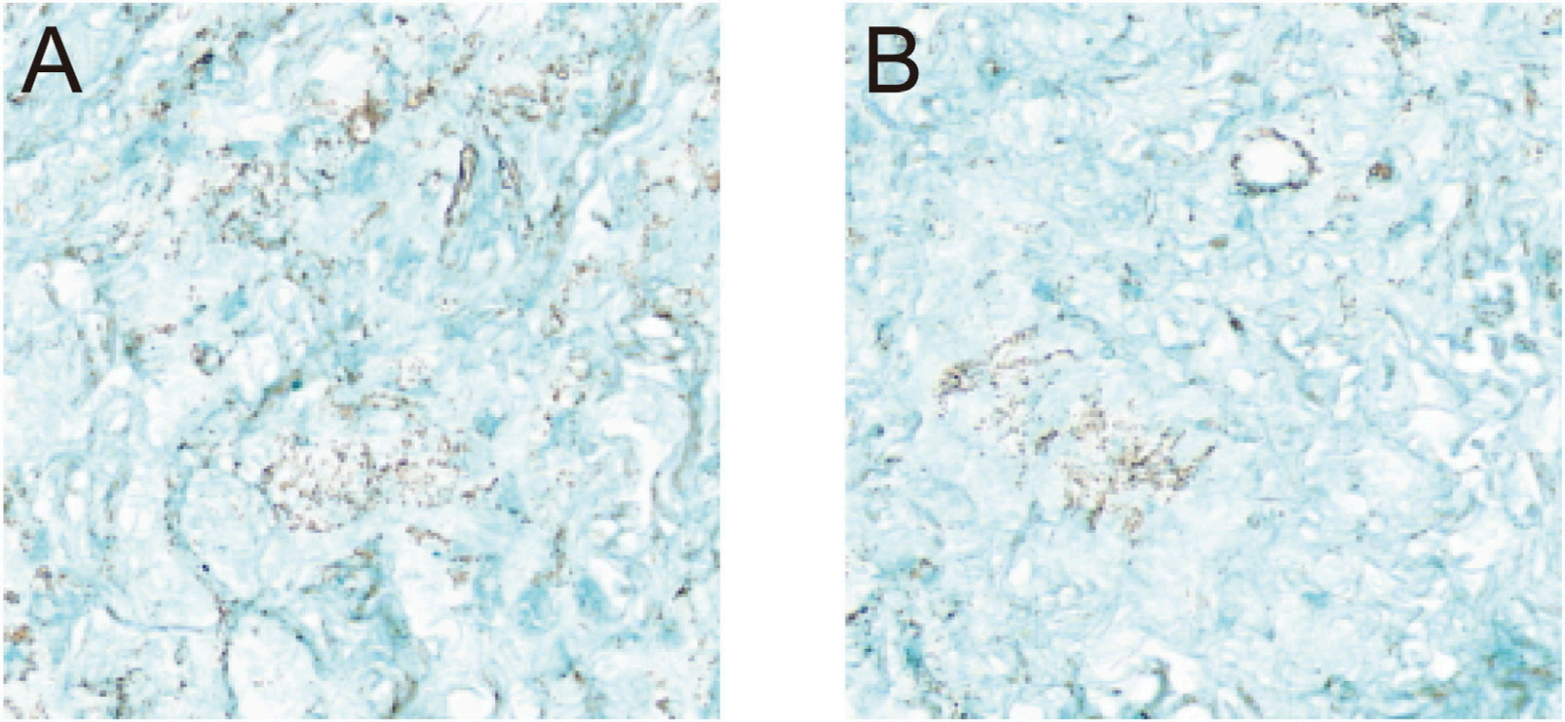
Chondrogenic Differentiation of BMSCs. Histological evaluation of BMSCs following 3 weeks of chondrogenic induction. **(A)** Unlabeled BMSCs at ×20 magnification; **(B)** USPIO-labeled BMSCs at ×20 magnification. Alcian blue staining revealed the formation of small, smooth, gel-like cartilage nodules in both groups, with no discernible differences observed between the unlabeled and labeled cells. These findings indicate that USPIO labeling does not affect the chondrogenic differentiation capacity of BMSCs.

Following 3 weeks of chondrogenic induction, both groups of BMSCs formed small, smooth, gelatinous cartilage-like spheroids. Histological sections of these spheroids, stained with Alcian blue, displayed prominent blue staining in both groups, confirming successful chondrogenic differentiation (Figure 5).

### 3.2 MRI evaluation of cartilage defect repair and T2 mapping analysis

**FIGURE 6:**
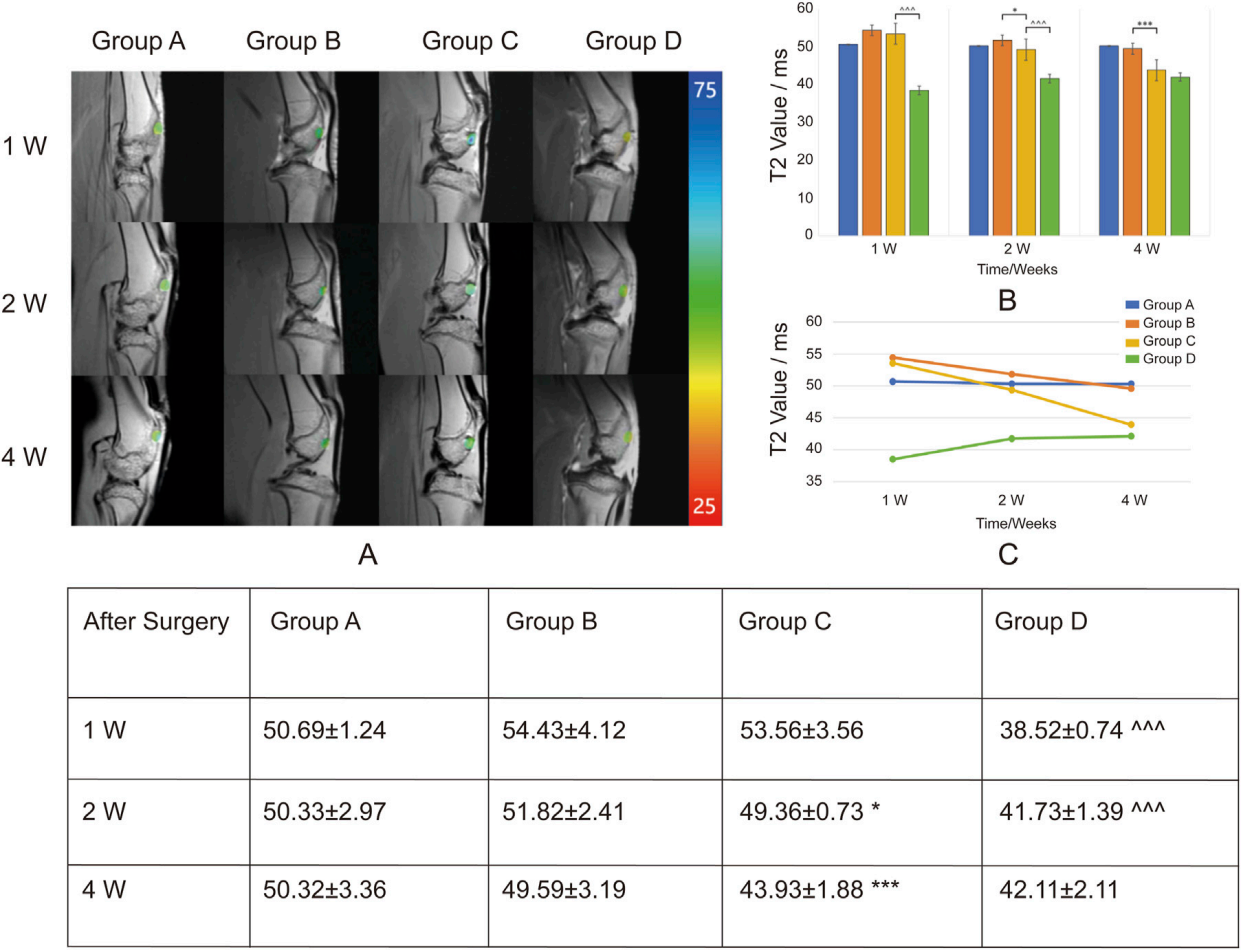
Overlay of T2 Mapping on Cartilage Defect Regions and T2 Values (ms) at Different Time Points. Group A: Control. Group B: Sodium alginate scaffold. Group C: BMSCs + sodium alginate scaffold. Group D: USPIO-labeled BMSCs + sodium alginate scaffold. **(A)** T2 mapping images overlaid on original MRI scans show the progression of cartilage repair in Groups A, B, C, and D at 1, 2, and 4 weeks post-surgery. The color scale represents T2 values (ms), ranging from 25 m (red) to 75 m (blue), illustrating changes in cartilage tissue composition during repair. **(B)** Bar chart displaying the mean T2 values (ms) in cartilage defect areas for Groups A, B, C, and D at 1, 2, and 4 weeks post-surgery. **(C)** Line chart showing the temporal trends of T2 values. Groups B and C demonstrate a continuous decline, indicating sodium alginate degradation and cartilage repair, with a more pronounced decrease in Group C. Group D shows a gradual increase in T2 values, suggesting ongoing cartilage regeneration and stabilization of the USPIO labeling effect. The table shows the Mean ± SD T2 values (ms) in the cartilage defect areas for Groups A, B, C, and D at 1 week (1w), 2 weeks (2w), and 4 weeks (4w) post-surgery. Significant differences are indicated as follows: B vs C (*P < 0.05, **P < 0.01, ***P < 0.001) and C vs D (^∧^P < 0.05, ^∧^^∧^P < 0.01, ^∧^^∧^^∧^P < 0.001).

T2 mapping post-processed images were superimposed on the original MRI scans to generate pseudocolor T2 mapping images of the cartilage defect regions for each group at different time points. Regions of interest (ROIs) were manually delineated, and the overlay images were obtained. T2 values in the cartilage defect regions of the rabbit knee joints were measured and compared using Functool software. (Figure 6). T2 values ranged between 25 and 75 ms, with pseudocolor changes from blue to green to red as T2 values decreased.

Group A was the blank control group, which did not receive scaffold implantation. T2 values at 1, 2, and 4 weeks post-surgery showed no significant changes, indicating a lack of notable cartilage repair and limited self-healing capacity of the cartilage.

In Group B, sodium alginate gel alone was implanted. The T2 values in the defect region decreased progressively at weeks 1, 2, and 4 post-surgery, suggesting gradual degradation of the sodium alginate gel. In Group C, BMSCs combined with sodium alginate gel were implanted. Compared with Group B, the T2 values in the defect region showed a more pronounced decrease over the same time points, indicating that BMSCs contributed to the repair process, potentially accelerating the degradation rate of sodium alginate gel.

In Group D, USPIO-labeled BMSCs combined with sodium alginate gel were implanted. The T2 values in the defect region were lower at all time points compared to Group C, reflecting the effect of USPIO as a superparamagnetic cell tracer that reduces T2 relaxation time. Additionally, the T2 values in Group D showed a gradual increase over weeks 1, 2, and 4, indicating that as the defect region underwent repair, the labeling effect of USPIO diminished and stabilized. This suggests that factors during the *in vivo* repair process may influence the effectiveness of USPIO labeling of BMSCs. At week 4 post-surgery, there was no statistically significant difference in T2 values between Group D and Group C, indicating that the USPIO signal had diminished by week 4. This suggests a limited tracking duration, making long-term tracking challenging.

### 3.3 Macroscopic evaluation of cartilage defect repair

**FIGURE 7:**
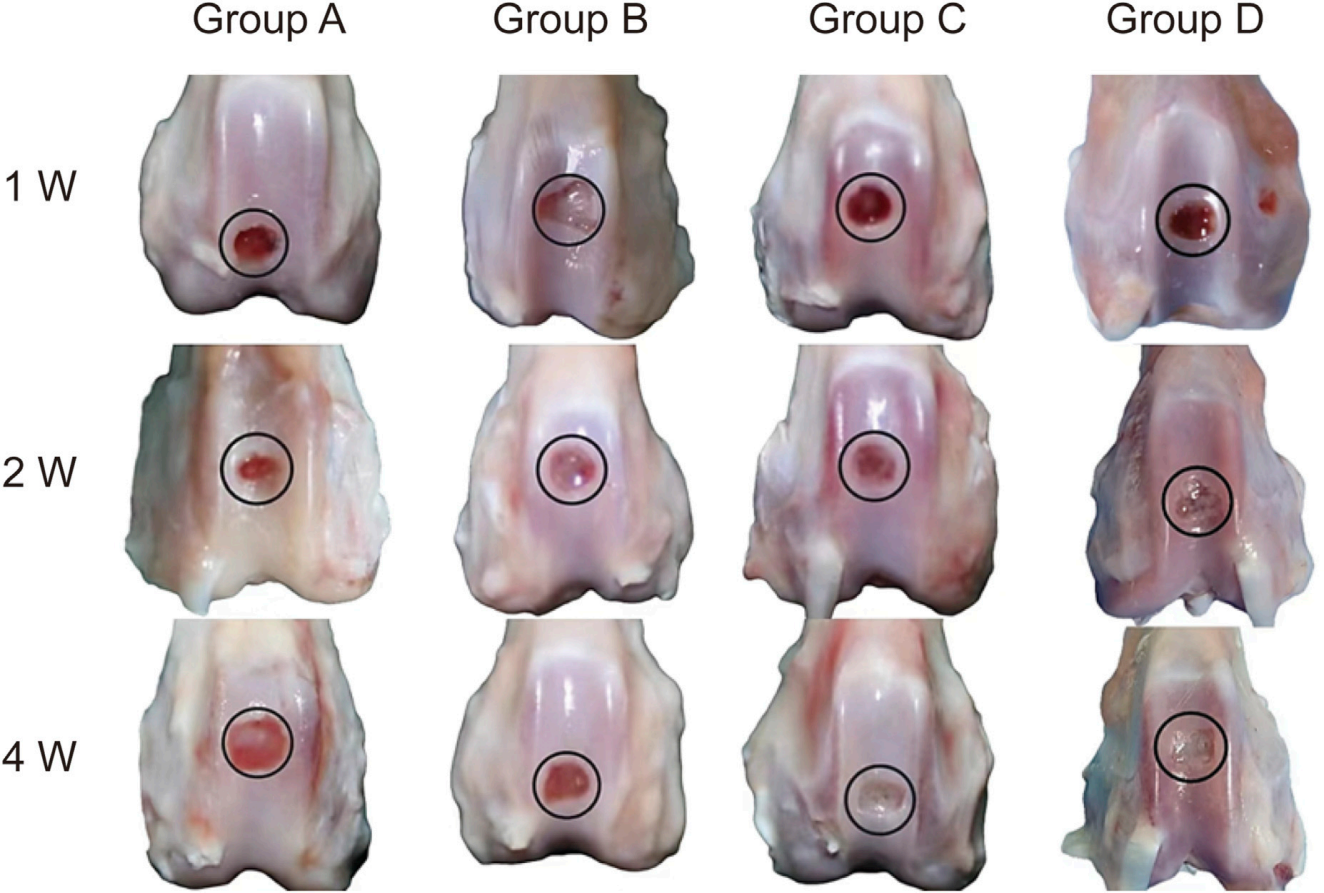
Gross Specimens of Cartilage Defect Repair in Knee Joints. Representative gross specimens showing cartilage defect repair in Groups A, B, C, and D at 1 week (1w), 2 weeks (2w), and 4 weeks (4w) post-surgery (Group A: Control. Group B: Sodium alginate scaffold. Group C: BMSCs + sodium alginate scaffold. Group D: USPIO-labeled BMSCs + sodium alginate scaffold). Group A displays minimal repair throughout the observation period. Group B shows slight tissue formation by 4 weeks. Groups C and D demonstrate progressive coverage of the defect with repair tissue, achieving approximately 80% coverage by 4 weeks. Both groups exhibit the most robust repair, with nearly complete filling of the defect area by 4 weeks.

Macroscopic examination of the femoral trochlear specimens at 1, 2, and 4 weeks post-surgery revealed the following findings: In Groups A and B, the defect regions remained recessed at weeks 1 and 2, with only minimal repair tissue observed by week 4. In contrast, Groups C and D exhibited small amounts of white repair tissue as early as week 1, which expanded in coverage by week 2. By week 4, the repair tissue covered approximately 80% of the defect area, with a mildly recessed surface and no visible sodium alginate gel residue. The repair outcomes in Groups C and D were comparable (Figure 7).

### 3.4 Histological evaluation of cartilage defect repair

**FIGURE 8:**
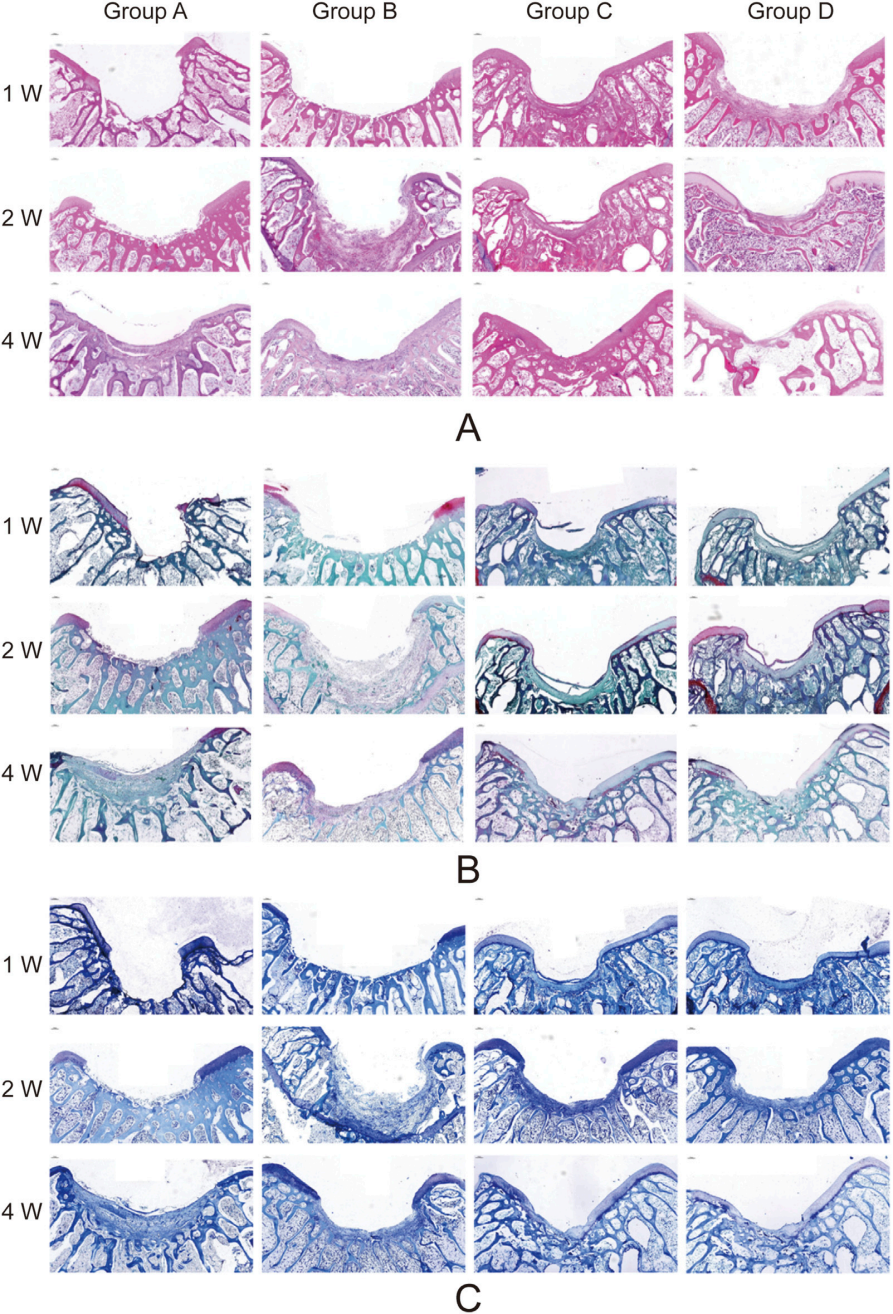
Histological Analysis of Cartilage Defect Repair in Knee Joints. Representative histological sections of cartilage defect areas in Groups A, B, C, and D at 1 week (1w), 2 weeks (2w), and 4 weeks (4w) post-surgery (Group A: Control. Group B: Sodium alginate scaffold. Group C: BMSCs + sodium alginate scaffold. Group D: USPIO-labeled BMSCs + sodium alginate scaffold). **(A)** Hematoxylin and Eosin (HE) staining shows tissue morphology and cellular distribution. Groups C and D demonstrate the most organized cartilage matrix with dense cell distribution by 4 weeks. **(B)** Safranin O-Fast Green staining highlights glycosaminoglycan deposition, with Groups C and D showing the highest content and most uniform distribution by 4 weeks. **(C)** Toluidine Blue staining further confirms the regeneration of the cartilage matrix, with Group C and D displaying superior cartilage repair compared to other groups. Groups B show moderate repair, while Group A exhibits minimal tissue formation.

Histological analysis using HE staining, Safranin O–Fast Green staining, and Toluidine Blue staining revealed the following findings: In Groups A and B, the defect regions were predominantly composed of fibrous tissue at weeks 1 and 2 post-surgery, with a small number of chondrocytes observed by week 4. In contrast, Groups C and D demonstrated early formation of cartilage matrix at week 1, with more organized matrix structures by week 2, and abundant chondrocytes filling the defect region by week 4. Groups C and D notably demonstrated comparable repair outcomes, exhibiting the most effective cartilage regeneration, with most defect areas filled by newly formed cartilage. The matrix showed significant restoration, with the highest glycosaminoglycan content and a uniform distribution (Figure 8).

## 4 Discussion

### 4.1 The key role of BMSCs in cartilage repair

This study highlights the significant role of BMSCs in promoting cartilage defect repair. Histological analysis revealed pronounced chondrocyte proliferation and matrix reconstruction at weeks 2 and 4 post-surgery, consistent with findings from numerous studies supporting the critical role of BMSCs’ remarkable multipotency in cartilage regeneration (Tan et al., 2021; Cong et al., 2023; Lv et al., 2023).

Furthermore, the study observed that BMSCs significantly accelerated the degradation of sodium alginate gel. This phenomenon may not only result from enzymes secreted by BMSCs but also involve physical interactions and remodeling mechanisms between cells and materials. For instance, BMSCs likely secrete matrix metalloproteinases (MMPs) to degrade sodium alginate gel directly. Simultaneously, the contraction and stretching forces exerted by BMSCs during growth and differentiation could alter the physical structure of the gel, indirectly promoting its degradation.

### 4.2 Comprehensive evaluation and optimization of sodium alginate as a scaffold material

Sodium alginate is widely utilized in tissue engineering due to its excellent biocompatibility and biodegradability (Chen et al., 2022). However, this study found that sodium alginate alone (Group B) demonstrated limited efficacy in cartilage repair, with T2 values consistently higher than those in Group C at all time points. This limitation may stem from its insufficient mechanical support in the early stages and its inability to effectively induce neocartilage formation during degradation.

When combined with BMSCs, the repair outcomes improved significantly, suggesting that sodium alginate’s primary advantage lies in providing a three-dimensional growth matrix that promotes cell adhesion, proliferation, and differentiation. Achieving optimal repair outcomes, however, requires synergistic interaction with bioactive cells or factors (Kang et al., 2023). To this end, the sodium alginate gel used in this study was supplemented with a complete medium containing fetal bovine serum, ensuring adequate nutrients for BMSCs proliferation and differentiation.

### 4.3 Impact of USPIO labeling on BMSCs

This study confirmed that USPIO labeling did not negatively impact the proliferation or chondrogenic differentiation capacity of BMSCs under experimental conditions, supporting its application as a cell tracer in cartilage repair research. BMSCs likely internalize USPIO through physical adsorption or endocytosis. At a dose of 25 mg Fe/kg, OD measurements revealed consistent proliferation curves between labeled and unlabeled groups, indicating good biocompatibility. Following chondrogenic induction, USPIO-labeled BMSCs successfully formed cartilage nodules, with Alcian blue staining confirming that their differentiation capacity remained unaffected.

### 4.4 Innovative application and challenges of combining T2 mapping and USPIO in monitoring cartilage repair

T2 mapping is widely used in cartilage imaging, as T2 values correlate with collagen fiber orientation and water content, providing a quantitative and visual assessment of structural changes during cartilage repair (Baum et al., 2013; Andreisek and Weiger, 2014; Hesper et al., 2014). In this study, T2 values in Groups B and C decreased at weeks 2 and 4 post-surgery, with a more pronounced decline in Group C at week 4. Macroscopic and histological analyses indicated sodium alginate degradation contributed to the T2 decrease, while BMSCs accelerated this process. Simultaneously, sodium alginate degradation promoted BMSCs chondrogenic differentiation, with the resulting cartilage matrix and chondrocytes further lowering T2 values. These findings suggest that T2 value changes reflect cartilage regeneration and sodium alginate degradation, with earlier decreases primarily indicating gel degradation and later decreases reflecting cartilage repair.

Previous studies indicate that BMSCs may undergo apoptosis post-transplantation due to harsh microenvironments and immune rejection, leading to repair failure. However, this study’s early-stage T2 value reductions were primarily associated with sodium alginate degradation, limiting accurate evaluation of BMSCs viability. By innovatively combining MRI with USPIO-labeled BMSCs, this study introduces a novel approach for *in vivo* BMSC tracking.

In Group D, T2 values were consistently lower than in Group C at weeks 1, 2, and 4, with a gradual increase during repair. Combined macroscopic and histological analyses suggest that as BMSCs proliferate and differentiate, T2 values slowly rise, indicating successful BMSC implantation and progressive cartilage defect repair. Studies have shown that the rapid disappearance of iron oxide signals at the transplant site correlates with cell death (Nejadnik et al., 2016; Theruvath et al., 2019). For instance, Nejadnik et al. transplanted live and dead USPIO-labeled hMSCs into osteochondral defects in athymic rats and observed faster T2 signal increases in the dead hMSCs group at days 14 and 28 (Nejadnik et al., 2016). These findings highlight the potential of this technique as a biomarker for assessing stem cell therapy efficacy.

Nevertheless, challenges persist. It is essential to acknowledge the inherent limitations of T2 mapping in assessing cartilage (Barbieri et al., 2024). Preclinical studies have highlighted several factors that can introduce measurement variability, including tissue heterogeneity—especially in transitional zones between regenerated and native cartilage—motion artifacts during image acquisition, and protocol variability across different imaging platforms (Baum et al., 2013; Barbieri et al., 2024; 2024). In this study, we employed several strategies to minimize these effects: (1) rigid fixation protocols to reduce motion artifacts in animal subjects, (2) standardized imaging parameters maintained across all time points, and (3) region-of-interest (ROI) analysis targeting central defect areas to mitigate heterogeneity-related variability. However, the small sample size (n = 6 per group) may limit the generalizability of the T2 value interpretations, a limitation commonly encountered in preclinical MRI studies. Future studies with larger cohorts and multi-center validation are needed to strengthen these findings. While USPIO labeling provides clear short-term cell tracking signals, its effects diminish over time (Sawah et al., 2022). In this study, T2 values plateaued by week 4, limiting its application for long-term monitoring. Potential causes include label dilution from cell division, intracellular metabolism of USPIO, and interference from the extracellular environment (Wei and Bao, 2022). Future research could combine USPIO with advanced imaging techniques to enhance long-term monitoring and explore more stable labeling strategies to overcome the challenges of slow cartilage regeneration.

## 5 Conclusion

In summary, this study achieved several key findings, demonstrating the synergistic application of USPIO-labeled BMSCs and sodium alginate scaffolds in cartilage defect repair. Through T2 mapping, the study enabled non-invasive, real-time monitoring of the repair process, with quantitative analysis of T2 value changes precisely tracking tissue remodeling and BMSCs dynamics. These findings provide an important assessment tool for clinical stem cell therapy.

## Data Availability

The raw data supporting the conclusions of this article will be made available by the authors, without undue reservation.
